# Studying hematopoiesis using single-cell technologies

**DOI:** 10.1186/s13045-017-0401-7

**Published:** 2017-01-21

**Authors:** Fang Ye, Wentao Huang, Guoji Guo

**Affiliations:** Center for Stem Cell and Regenerative Medicine, Zhejiang University School of Medicine, Hangzhou, 310058 China

**Keywords:** Single-cell analysis, Hematopoietic stem cell, Lineage hierarchy, Regulatory network

## Abstract

Hematopoiesis is probably the best-understood stem cell differentiation system; hematopoietic stem cell (HSC) transplantation represents the most widely used regenerative therapy. The classical view of lineage hierarchy in hematopoiesis is built on cell type definition system by a group of cell surface markers. However, the traditional model is facing increasing challenges, as many classical cell types are proved to be heterogeneous. Recently, the developments of new technologies allow genome, transcriptome, proteome, and epigenome analysis at the single-cell level. For the first time, we can study hematopoietic system at single-cell resolution on a multi-omic scale. Here, we review recent technical advances in single-cell analysis technology, as well as their current applications. We will also discuss the impact of single-cell technologies on both basic research and clinical application in hematology.

## Background

The common landscape of cellular hierarchy has been depicted as a transitional process through multiple intermediate states. In mammalian systems, many stem and progenitor cell types have been identified with a combination of cell surface markers, analyzed through multicolored fluorescence-assisted cell sorting (FACS). In hematopoietic system, extensive efforts have gone into the characterization of cellular differentiation pathways and genetic regulatory network [1]. The identification of mouse hematopoietic stem cell and progenitor cells (HSPC) [2, 3], along with the separation of hematopoietic stem cells from multipotent progenitors (MPP) [4–7], has indicated that multipotent progenitors (MPP) are generated from self-renewing hematopoietic stem cells. Further identification of common lymphoid progenitors (CLP) and common myeloid progenitors (CMP) [8, 9] suggested that CLP and CMP stand at the bifurcation point of this lineage model. However, other recent findings challenged this view, since they found heterogeneity within these classical progenitors, which implied alternative lineage commitment pathways [10–12].

The signaling pathways and transcriptional networks that regulate hematopoietic stem cell emergence, self-renewal, and differentiation are not well understood [1, 13]. Previous works utilized FACS to separate and purify different progenitor types, and then performed transcriptomic analysis on each cell population. Those works elucidated a complex transcriptional network in the hematopoietic hierarchy [14, 15]. However, these analyses were conducted on bulk samples, which neglected the heterogeneity in the defined population as well as unknown transitional states during the cell fate decision process.

Not until recent years, single-cell analysis has become a powerful tool for studying cellular differentiation pathways. Advances in technology made it possible to track and capture single cells, then analyze its genome, transcriptome, and proteome. For instance, high-throughput single-cell quantitative PCR is highly sensitive in detecting quantitative differences [16–18]. Single-cell RNA-seq [19–22] allows for transcriptome analysis. High-throughput single-cell barcoding and sequencing approaches [23–26], coupled with data analysis methods [27, 28], enable the dissection of heterogeneity in complex cellular systems. In the field of hematopoietic study, single-cell transcriptomic and proteomic analysis provide unprecedented insights into cellular differentiation hierarchy, gene regulatory network, and developmental origin as well as mechanisms for stem cell aging. In this paper, we review the recent technical advances in single-cell methodology and summarize their features and contribution to hematopoietic research. In the end, we will discuss the current challenges and future directions in the field.

## Technical advances in single-cell analysis

The cellular heterogeneity is concealed in the analysis of bulk cells. Cells gathered from the same part of the tissue differ from each other in gene expression and epigenetic status. The single-cell analysis provides a solution to understand the heterogeneity within cell population. Recently, technical developments have been made in the amplification of rare nucleic acid templates (Table 1).

**Table 1:** Classification of single-cell analysis methods

Method	Amplification	Coverage	References
Genomics			
MDA	MDA	High coverage	[30]
MALBAC	MALBAC	High coverage	[31]
Transcriptomics			
Single-cell qPCR	Multiplexed PCR	Target gene	[16]
Tang-seq	PolyA tailing + second-strand synthesis	Full-length mRNAs	[34]
CEL-seq	In vitro transcription	3′ End of mRNA	[21]
Smart-seq	Template switching	Full-length mRNAs	[36]
Cyto-seq	Multiplexed PCR	3′ End of mRNA	[23]
Drop-seq	Template switching	3′ End of mRNA	[24]
inDrop	In vitro transcription	3′ End of mRNA	[25]
Proteomics			
Mass cytometry	NA	Target protein	[55]
Epigenomics			
scATAC-seq	Adaptor PCR	Accessible DNA regions	[64]
scRRBS	Adaptor PCR	1.5 million CpG sites	[59]
scHi-C	Adaptor PCR	NA	[63]
scChIP-seq	Adaptor PCR	About 1000 peaks	[62]

*NA* not applicable

### Single-cell genomic methods

Several single-cell whole genome amplification (WGA) methods have been developed to amplify the rare genomic DNA. Degenerate-oligonucleotide PCR was used for analyzing copy number variation in cancer cells [29]. Another well-known WGA method was multiple displacement amplification (MDA), which utilized random primers and bacteriophage polymerase to achieve high-coverage single-cell exome sequencing [30, 31]. Multiple annealing and looping-based amplification cycles (MALBAC) have also been developed to reduce the bias in nonlinear genome amplification process. MALBAC achieves both high-coverage and uniform amplification. It can be applied to detect both copy number variations (CNVs) and single nucleotide polymorphisms (SNPs) in single-cell genome [32].

### Single-cell transcriptomic methods

**Fig. 1:**
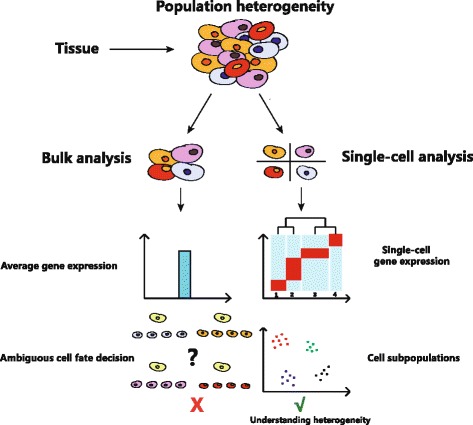
Single-cell analysis reveals heterogeneity. Traditional experiments on bulk samples mask the heterogeneity between individual cells. In order to understand the heterogeneity in complex tissue, analysis performed on single-cell resolution has been used to unveil cell subpopulations and their different gene expressions

Single-cell transcriptome analysis remarkably serves as a powerful tool for studying cellular heterogeneity and lineage hierarchy (Fig. 1). There are several available methods: single-cell qPCR [16], single-cell microarray analysis [33], and single-cell RNA-seq [34, 35]. After single-cell isolation from complex tissue, the first challenge is to amplify the small amount of RNA, which is about 10 pg per cell. Four mainstream strategies are used: multiplexed RT-PCR, polyA tailing followed by second-strand synthesis [19], template switching, and in vitro transcription (IVT) [21]. Multiplexed RT-PCR is used in single-cell qPCR experiment [16]. Single-cell qPCR does not need to sequence the sample. It is convenient for detection of dozens of genes. PolyA tailing method was used in single-cell microarray and Tang-seq studies. Smart-seq and Smart-seq2 amplification is a widely used approach for the full-length mRNA analysis of single cells [22, 36, 37]. It uses the template-switching-based protocol to append a primer binding site on the 3′ end of the cDNA. cDNA is then amplified by PCR and sequenced by Illumina sequencing platform. The mRNA coverage of Smart-seq is between 10 and 20%. IVT used in CEL-seq and MARS-seq accomplishes a linear amplification of RNA using T7 promoter and RNA polymerase [21, 38]. The unique molecular identifiers (UMIs) are designed for reducing the amplification bias [39]. They enable the absolute counting of mRNA molecules in the single cell when mRNA capture efficiency and the sequencing depth are good enough. The low coverage of mRNA is a common problem for all existing methods.

Recently, application of single-cell transcriptomic analysis has rapidly spread to many areas such as early embryonic development [16, 40–44], cellular reprogramming [18, 45], human breast cancer [46], metastatic melanoma [47], circulating tumor cells [48], olfactory neurogenesis [49], early embryo development [50], neuronal cell heterogeneity, and immune cell pathogenicity [51–53]. These applications demonstrate the broad applicability of single-cell transcriptomic analysis.

### Single-cell proteomic methods

Traditional single-cell protein analysis depends on fluorescence flow cytometry [54]. The development of mass flow cytometry notably increased multiplexity by isotope label on antibodies [55]. This method resolved the spectral overlap problem in fluorescence flow cytometry and can detect more than 30 parameters simultaneously. The idea has also been used in multiplexed ion beam imaging (MIBI) [56], which is capable of analyzing up to 100 targets at the same time in the tissue sections. Recent advances in microfluidic chips also enabled multiplexed analyses for quantitative single-cell proteomics [57, 58]. All existed methods only allow detection of limited kinds of protein. A whole proteome analysis approach remains to be developed.

### Single-cell epigenomic methods

Single-cell epigenomic technologies are becoming more and more accessible. Single-cell reduced representation bisulfite sequencing (scRRBS) and single-cell 5hmC-sequenceing were applied to investigate DNA methylation [59–61]. Single-cell chromatin immunoprecipitation sequencing (ChIP-seq) [62] and single-cell Hi-C [63] have been developed to profile chromatin structure in single cells. Single-cell chromatin accessibility methods, such as single-cell assay for transposase-accessible chromatin with high-throughput sequencing (ATAC-seq), have been used to investigate cell-to-cell variation in mammalian regulatory elements [64]. Large-scale profiling of single-cell chromatin accessibility landscape can be achieved by combining cellular indexing and ATAC-seq [65].

### Single-cell capture methods for sequencing

**Fig. 2:**
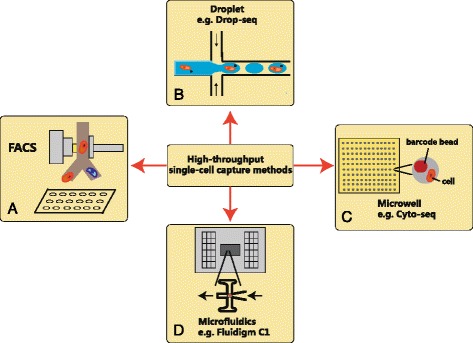
High-throughput single-cell capture methods. **a** FACS sorting using monoclonal antibodies. **b** Microfluidic droplet generation. **c** Microwell captures single-cell and barcode bead simultaneously by gravity. **d** Fluidigm C1 single-cell platform based on large-scale microfluidic system

Single-cell capturing is a challenge. However, we have seen the significant progress of platform developments in recent years (Table 2). When samples are rare, mouth pipetting and laser capture microdissection (LCM) [66] are good choices to isolate single cells. But when dealing with large number of cells, throughput becomes the bottleneck. FACS played an importing role in scaling up single-cell collection efficiency, but library generation remains to be labor intensive and costly. Very recently, lots of other convenient methods have been invented (Fig. 2). Some of them are directly linked with combinatorial indexing and sequencing library generations, which greatly facilitated high-throughput single-cell analysis without requirements for expensive instruments.

**Table 2:** The advances of single-cell capture methods

Methods	Advantage	Drawback	Application
Mouth pipetting	Low cost	Time consuming	Rare sample
Laser capture microdissection	Visualization	Time consuming	Specific target
Flow cytometry	Marker selection	Require sorting	MARS-seq
Microwell platform	High throughput	mRNA capture rate	Cyto-seq
Microdroplet platform	High throughput	mRNA capture rate	Drop-seq, inDrop
Fluidigm C1 platform	Automatic library prep	High cost	qPCR, mRNA-seq
DEPArray	Visualization	High cost	Specific target

#### Fluidigm C1 system

Fluidigm C1 system, a commercialized single-cell library preparation platform, uses microfluidic circuits for single-cell capture and mRNA amplification reaction [67]. The whole work flow is highly automatic, and its data shows good stability. It becomes a widely recognized platform for single-cell analysis [68–71]. The high cost for the device and consumables is its main limitation.

#### DEPArray

DEPArray is another commercialized imaging-based cell sorting platform, which is suitable for selecting rare cells from sample. First, cells are loaded to a microfluidic cartridge, which contains an array of individually controllable electrodes. Then, every individual cell is trapped in a dielectrophoresis (DEP) cage. After imaging, cells of interest can be moved into parking chamber and recovery chamber. This technique allows isolation of tumor cells from the tissue and blood [72] and collection of circulating tumor cells (CTCs) [73, 74]. The ability to image and manipulate individual cell is the main feature of DEPArray. Rare cells can be identified, recovered, and sequenced subsequently.

#### Cyto-seq

The Cyto-seq method uses a microwell array to capture a large number of single cells [23]. Individual cell and indexed bead are trapped in the same well. After cell lysis, mRNAs are captured by indexed oligonucleotides on beads. The beads are then pooled, followed by a reverse transcription. The indexed cDNAs are then amplified with primers of target genes using multiplexed PCR. Expression of targeted genes is quantified by unique molecular identifiers (UMIs). The microwell array used by Cyto-seq is very simple to fabricate. The system is relatively convenient for small labs to set up.

#### Drop-seq and inDrop

One notable high-throughput platform developed very recently is the droplet-based microfluidics [26], which can efficiently sort individual cells into thousands of nanoliter droplet. The nanoliter droplet serves as a tiny reaction chamber, which significantly reduces the usage of reagent and increases the concentration of target molecules. In 2015, two groups simultaneously applied the droplet-based microfluidics single-cell analysis platforms to large-scale transcriptome study, which are termed Drop-seq and inDrop, respectively. Drop-seq puts individual cell and an indexed bead into a nanoliter droplet, where each bead captures the mRNAs from single cell. Then, the beads are pooled, and mRNAs can be reverse transcribed. The indexed cDNAs from thousands of cells are together amplified and sequenced [24]. Meanwhile, inDrop demonstrates a very similar strategy, but the reverse transcription reaction is done inside the droplets. It utilizes IVT rather than template switching method for the transcriptomic amplification [25]. Both approaches adopted UMIs to quantify individual mRNAs. However, the dramatic increase of throughput comes with a cost. The detected gene number for single cells drops down due to the limitation of sequencing depth.

### Single-cell imaging

Single-cell imaging enables phenotypic characterization of single cells, while preserving their spatial information. DEPArray has image-based single-cell sorting function. Besides, time-lapse microscopy allowed continuous live image on single cells. This method provides important information of the dynamic cell fate decision process during blood cell differentiation [75, 76]. Hardware and software requirements for setting up the single-cell long-term imaging system have also been thoroughly discussed [77].

### Single-cell transplantation

Single-cell transplantation is a powerful approach to verify the stem cell identity. Single-cell transplantation of hematopoietic stem cell (HSC) purified by different surface marker proved their multilineage reconstitution function [5, 78]. Recently, Notta et al. revealed CD49f as a human HSC marker. Flow-sorted single cell based on CD49f and mitochondrial dye rhodamine-123 displayed robust chimerism even 20 weeks after transplantation [79].

### Single-cell data analysis

The next-generation sequencing platforms generate massive data set. The large amount of cell and gene dramatically increases the dimension and complexity of the data. Traditional computational methods are no longer suitable. Several single-cell computational methods have been developed. Here, we briefly introduce the workflow of single-cell data analysis.

#### Data preprocessing

Single-cell sequencing generates huge amount of data. In single-cell transcriptomic sequencing analysis, lots of computational tools are developed for preprocessing, normalization, and transcripts quantification. A recent review gave a standard pipeline of handling single-cell RNA-seq data with or without UMIs [80]. For single-cell RNA-seq, spike-in RNA is recommended as an artificially designed internal controller in experiments to estimate technical variation [81, 82]. After filtering out data with low quality, sequencing reads would be aligned to a reference genome or transcriptome. After counting and normalization, the single-cell transcriptomic data will be converted into a digital gene expression (DGE) matrix for further analysis. For single-cell genomic data, people developed normalization algorithms based on channel, genome composition, and recurrent genome artifact corrections to improve the CNV detection in single-cell array CGH data [83]. An optimized protocol was used to correct biases inherent in the WGA procedure for the genome-wide copy number analysis [84]. For single-cell qPCR data processing, the use of a single reference gene is not recommended. In order to address the inherent noise in single-cell gene expression data, normalization by the median Ct value was applied [85]. For mass cytometry, bead based signature and an algorithm was used to determine data quality [86].

#### Data visualization and clustering

The downstream analysis focuses on visualizing the high-dimensional single-cell gene expression data and clustering the transcriptionally distinct subgroups. One visualization tool for high-dimensional data is principal component analysis (PCA), which has been widely applied in single-cell research [16, 25, 87]. PCA maps the high-dimensional data points into a low-dimensional space. Another visualization tool for reducing high-dimensional data into two or three dimensions is *t*-distributed stochastic neighbor embedding (t-SNE) [88, 89]. In SNE, nearby data points in high-dimensional space remain their similarity in low-dimensional space. However, SNE is hampered by the crowding problem, which means that the clusters could not be totally separated from each other. To alleviate the crowding problem, the Student’s *t* distribution method is introduced in t-SNE to compute the similarity between two points. As a powerful visualization tool, it has demonstrated great capacity in recent high-throughput single-cell studies [24, 47]. PCA and t-SNE are usually combined for the visualization of large-scale data. After identification of cell subpopulation, one can extract specific gene markers for each subpopulation. To improve differential gene identification from noisy single-cell data, Kharchenko et al. reported a probabilistic model of expression-magnitude distortions typical of single-cell RNA-sequencing measurements [90]. Other downstream clustering pipelines for high-throughput single-cell gene expression are mostly based on R package or MATLAB. A computational strategy named Seurat integrated these visualization methods and tools into an R package to deal with single-cell RNA-seq data [24, 91].

#### Pathway and network modeling

During lineage commitment process, the continuity of single-cell gene expression can be used to infer differentiation pathway. Spanning-tree progression analysis of density-normalized events (SPADE) analysis uses this idea to infer cellular hierarchy from large-scale single-cell data set without assigning temporal order [17, 55, 92, 93]. For gene regulatory network, some research groups completed network modeling with STRING and functional NET database [94, 95]. Weighted gene co-expression network analysis (WGCNA) is a R package which is available for conducting weighted gene co-expression network analysis [96]. Gene expression networks could also be integrated with epigenomic data like ChIP-seq binding data sets [17]. Computational tools have strengthened our ability to extract valuable information from large-scale data, thereby playing an indispensable role in the single-cell analysis.

## Studying hematopoiesis at single-cell level

Classical knowledge about hematopoiesis is built on cell type definition system using flow cytometry analysis. Such a system is limited with a small number of cell surface markers for cell classification. Recently, taking the advantage of the aforementioned technologies and the related data analysis pipelines, we start to realize that many of these classical cell types are heterogeneous. Single-cell analysis helped to provide unprecedented insights into long-lasting questions in topics of hematopoietic study, such as HSC heterogeneity, differentiation pathway, fate decision, regulatory network, HSC aging, and HSC origin.

### HSC heterogeneity

Blood cell production depends on HSC’s self-renewal and multilineage differentiation abilities. However, hematopoietic stem cells are heterogeneous in differentiation behavior. Single-cell transplantation is the most definitive assessment of HSC functional heterogeneity. Classical single-cell transplantation experiment showed that HSC defined by mouse homolog of CD34 reconstituted the lymphohematopoietic system for more than 3 months in mice. Highly purified mouse HSCs based on the expression of CD34 demonstrate variability in self-renewal potential and multilineage differentiation potential [5]. A recent research utilized single-cell transplantation assay to analyze phenotypic long-term HSC systematically. Donor-derived contribution to the circulating white blood cells showed at least four distinct patterns. They provide solid evidence that primitive hematopoietic cells can maintain distinct repopulation properties upon serial transplantation in vivo [97]. Using similar approaches, Morita et al. found that in the HSC subset, single cells behave differently based on their CD150 expression. Decreased expression of CD150 appears to be associated with reduced erythroblast/megakaryocyte differentiation potential. The balanced long-term repopulating cells are enriched in the CD150 intermediate subpopulation [78]. To gain deeper insight into the regulatory program of mouse HSCs, Wilson et al. linked single-cell functional assays with flow cytometric index sorting and single-cell gene expression assays. They identify key molecules that associate with long-term durable self-renewal and provide a single-cell molecular dataset that can be further analyzed regarding HSC heterogeneity [98].

### Differentiation pathway

**Fig. 3:**
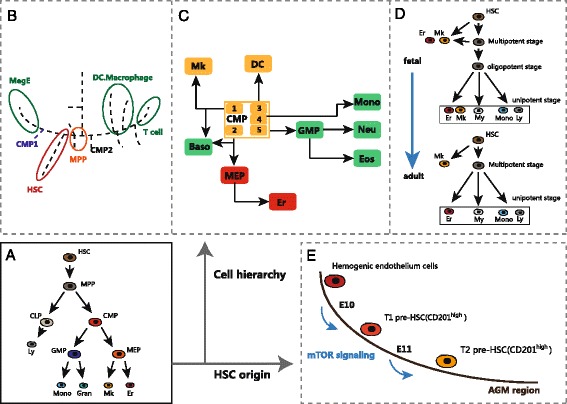
New findings on hematopoietic hierarchy and origin of hematopoietic stem cell by single-cell analysis. **a** Traditional step-down cell hierarchy model. **b** Single-cell SPADE hierarchy demonstrate early separation of MegE and lympho-myeloid lineage. **c** Transcriptional heterogeneity and cell hierarchy in myeloid progenitor populations. **d** Redefined model demonstrates two different development stage in the progenitor cell. **e** Tracing pre-HSC at single-cell level

In the classical model of hematopoiesis, an organized hematopoietic lineage tree starts with multipotent HSC, and then followed by oligopotent and unipotent progenitors. However, recent single-cell results challenged the classic model and proposed that traditional hematopoietic progenitor types are very heterogeneous [99, 100]. Guo et al. used 280 multiplexed qPCR assays to analyze over 1500 single mouse hematopoietic cells [17]. The analysis revealed dramatic heterogeneity within all of the classically defined progenitor types, such as HSC, MPP, CMP, and CLP. The comprehensive data revealed a revised hierarchy of hematopoietic stem cell differentiation in which megakaryocytic and erythroid (MegE) lineage was the first to branch from hematopoietic stem cells (Fig. 3b). More recently, Notta et al. combined single-cell gene expression analysis and single-cell functional assay to study human hematopoiesis, and their results challenge the classical human hematopoietic hierarchy model [100] (Fig. 3d). They found that the cell hierarchy differed from fetal stage to adult stage. In fetus, multipotent, oligopotent, and unipotent progenitors are all can be seen, while only multipotent and unipotent progenitor stages were observed in adult. They show that megakaryocytic lineage can derive from HSC and multipotent progenitors in fetus but only branch from HSC in adult. Franziska et al. combined index sorting with MARS-seq to analyze mouse bone marrow CMP. The remarkable data set revealed seven transcriptionally distinct subpopulations within CMP cells. These subpopulations showed unexpected priming towards seven differentiation fates but no progenitors with a mixed state. The findings challenged traditional common myeloid progenitors (CMP) defined by cell surface markers and built a single-cell reference for studying mouse myeloid differentiation [99] (Fig. 3c). Kristiansen et al. analyzed the differentiation process from fetal liver HSCs to B-1a/B-2 B cells and provided novel insights into the B cell lineage development [101]. Macaulay et al. applied single-cell RNA-seq to study thrombocyte lineage commitment in zebra fish. They placed all data points into a continuum to form a refined lineage pathway [102]. Another work combined single-cell transcriptional profile and immune-phenotype to clarify differentiation pathway in megakaryocyte-erythroid progenitors (MEP) [103].

Single-cell mass cytometry is also a widely used method in hematopoietic differentiation pathway study. A recent primer article detailed the development of mass cytometry and related data analysis methods [104]. Bendall et al. measured 34 parameters for human bone marrow samples. Using the data, they constructed a minimal spanning tree-based hematopoietic differentiation pathway. The research also revealed system-wide signaling responses among traditional cell subsets [55]. Single-cell mass cytometry was also used to map cell cycle phases for human hematopoietic cells [105]. The method allowed deep profiling of major phases of the cell cycle simultaneously in single cells. Single-cell mass cytometry data can be aligned onto a unified trajectory. This approach accurately predicts the stepwise human B cell developmental path de novo. The trajectory revealed virtually all the cellular states of early B cells differentiated from hematopoietic stem cell [106].

Altogether, these findings challenged the current step-by-step bifurcation hierarchy in hematopoietic system. Transcriptional heterogeneity among progenitors suggested that cell differentiation may proceed in a more sophisticated way. Single-cell analysis helped to rebuild hematopoietic lineage hierarchy and identify markers of previously unknown subpopulations.

### Fate decision

How do blood cells make their lineage decisions? Single-cell analysis helped to resolve this question with higher precision. Pina et al. used single-cell qPCR to define primary multipotent self-renewing cells and early erythroid-committed cells [107]. Their data suggested an uncoordinated molecular transition between self-renewal and committed states. They also found dissociation between self-renewal potential and transcriptome-wide activation in lineage program. Multipotent cells are unlikely to change into the committed state under independent activation by individual regulators. A very recent work aimed to capture mixed-lineage states in mouse hematopoietic stem and progenitor cells. With single-cell transcriptomic data, they provided evidence that mixed-lineage intermediates manifested concurrent expression of hematopoietic stem cell/progenitor and myeloid progenitor cell genes [108].

Single-cell imaging and tracking methods are also powerful tools for examining blood generation and lineage determination. Eilken et al. used time-lapse microscopy to achieve continuous long-term single-cell observation and detected hemogenic endothelial cells giving rise to blood cells [75]. Hoppe et al. applied time-lapse imaging and single-cell tracking to explore co-regulation of transcription factors GATA1 and PU.1 in differentiation dynamics of single HSCs [76]. They found that the ratio of PU.1 and GATA1 is not a key regulator for HSPC lineage decision, which challenged the old view on the early hematopoietic lineage separation.

### Regulatory network

The explosion of genomic data enabled generation of regulatory networks in various biological systems. However, network build with data from population of cells are intrinsically flawed, because the fundamental unit of gene regulation is single cell, rather than heterogeneous population. Regulatory model at single-cell level starts to emerge in recent years. In 2013, Moignard et al. analyzed 18 key hematopoietic transcription factors in hundreds of blood stem cells and progenitor cells [109]. They revealed factor interaction between Gata2, Gfi1, and Gfi1b. In their model, Gfi1 represses Gata2, whereas Gata2 activates Gfi1b. Gata2 functions in a regulatory loop to modulate Gfi1/Gfi1b cross-antagonism during HSC entry into the lympho-myeloid lineages. In the same year, Guo et al. build a remarkably similar single-cell model to explain early megakaryocyte and erythroid lineage regulation, in which Gata2 primes MegE fate and represses lympho-myeloid fate [17]. In 2015, Moignard et al. introduced another strategy using diffusion maps to analyze single-cell qPCR data. Based on state transition graphs from 3934 cells in the mouse embryo, they generated a transcriptional regulatory network model to explain the whole blood development process [28]. Thanks to the emergence of high-throughput single-cell analysis method, we are able to elucidate gene regulation mechanisms for the first time at the single-cell level.

### HSC aging

It has been a debate whether intrinsic cell changes or variations in composition contributes to the systematic HSC aging. Single-cell analysis helped to provide a deeper look into this question. Grover et al. analyzed old and young HSCs transcriptomes at the single-cell level. They identified significantly increased molecular platelet priming and functional platelet bias in the aged HSCs [110]. They observed that loss of the FOG-1 transcription factor associated with HSC platelet programming increased lymphoid output. Thus, increased platelet bias is a key process during HSCs ageing. Kowalczyk et al. compared cells from young and old mice by single-cell RNA-seq. They found lower frequency of cells in G1 phase among old long-term HSCs. Moreover, old short-term (ST) HSCs resemble young long-term (LT) HSCs, suggesting that they exist in a less differentiated state [111]. Single-cell analyses of HSC aging process demonstrate that both compositional changes and intrinsic, population-wide changes contribute to phenotypic aging.

### HSC origin

The origin of HSCs in mammalian systems has long been a mystery. The classical study identified the primitive type of hematopoietic stem cell in yolk sac (YS-HSC). But definitive hematopoiesis is maintained by HSC originated within the aorta-gonad-mesonephros (AGM) region of the embryo [112, 113]. Definite hematopoiesis produces HSCs with multilineage potential and long-term reconstitution ability. A recent work adopted single-cell RNA-seq to analyze endothelial cells and pre-HSCs in the mouse AGM region. Zhou et al. demonstrated that pre-HSCs have unique features in transcription factor network, signaling pathway, and unique cell cycle status at the single-cell level. They identified new surface markers for pre-HSC isolation and revealed the importance of mTOR in regulating the stepwise generation of HSCs in vivo (Fig. 3e). Transplantation of isolated pre-HSCs demonstrates strong self-renewal capability. New marker of pre-HSCs would provide new insights into HSC development and push forward future clinical applications of HSCs [114, 115]. Thus, single-cell analysis show great convenience and precision in tracing the origins of HSCs. Future efforts may focus on the transition of HSC heterogeneity during developmental stage. Besides, the origin of human hematopoietic stem cells also deserves further investigation.

## Conclusions

Single-cell analyses have achieved remarkable advancement in recent years. Hematological researches also benefited from the rapid progress of single-cell technology. Future application of these approaches will impact the field in many aspects.

One important direction for single-cell analysis is to develop cost-effective methods. The present commercialized single-cell analysis equipment is limited by both cost and throughput. More economical and convenient methods are required for broad application of high-throughput single-cell analysis. As technology goes, future throughput is expected to achieve tens of thousands of single cells per experiment, while the cost is going to drop below 0.1 USD per cell.

Another orientation is the large-scale single-cell database. The explosively increasing single-cell data has raised a big challenge for scientists. Data comparison between different studies remains to be difficult. Data management becomes a burden for ordinary labs. Better computational analysis methods are needed. A large-scale database and online data analysis pipeline would be extremely helpful for the integration of various data sets collected from different tissues. An open-access platform may eventually lead to the completion of human single-cell atlas database, which might significantly impact basic research and clinical diagnosis.

In the future, multi-omics will be a trend for single-cell analysis, such as profiling DNA methylome and transcriptome from the same cell simultaneously [116]. Single-cell triple omics sequencing method, including genome, DNA methylome, and transcriptome, is another innovation [117]. A complete circuit of a single cell can be detected by the integration of genomics, epigenomics, transcriptomics, and proteomics. Multi-omics data will provide a more comprehensive understanding to the hematopoiesis and other basic questions of life.

In situ single-cell sequencing methods will preserve spatial information. It should be extremely helpful for studying stem cell microenvironment. Cell niches are playing essential roles for the self-renewal and differentiation of hematopoietic stem cell in vivo. Synthetic microenvironment has been used to generate functional hematopoietic stem cells [118–120]. In situ single-cell genomics would be ideal to analyze hematopoiesis in their local niches. Such information should be important for understanding the behavior of HSC and leukemia. It will provide guidance for promoting HSC expansion and to inhibit leukemia growth.

The progress of basic methodologies will accelerate the diagnosis and treatment for hematological diseases. Single-cell analysis of wild-type blood cells may offer guidance for HSC generation in vitro and improve transplantation-based therapies. Single-cell analysis of leukemia cells will help to find heterogeneity and clonal composition in cancers and guide leukemia treatments [121]. Recently, the grand plan for precision medicine has been put on agenda. Nothing is more precise than single cells. Single-cell analysis will allow for highly precise treatment for different individuals, as well as different cells in one patient. This powerful strategy will have a long-term impact on both basic research and clinical application in the field of hematology.

## Abbreviations

AGM: Aorta-gonad-mesonephros
ATAC-seq: Assay for transposase-accessible chromatin with high-throughput sequencing
Baso: Basophil
CLP: Common lymphoid progenitors
CMP: Common myeloid progenitors
CNVs: Copy number variations
CTCs: Circulating tumor cells
CyTOF: Cytometry by Time-Of-Flight
DC: Dendritic cells
DEP: Dielectrophoresis
DGE: Digital gene expression matrix
E: Embryonic days
Eos: Eosinophil
Er: Erythrocytes
FACS: Fluorescence-assisted cell sorting
GMP: Granulo-monocyte progenitors
Gran: Granulocytes
Hi-C: High-throughput/resolution chromosome conformation capture
HSC: Hematopoietic stem cell
HSPC: Hematopoietic stem and progenitor cells
IVT: In vitro transcription
LCM: Laser capture microdissection
Ly: Lymphoid
MALBAC: Multiple annealing and looping-based amplification cycles
MATLAB: Matrix laboratory
MDA: Multiple displacement amplification
MegE: Megakaryocytic and erythroid
MEP: Megakaryocyte-erythroid progenitors
Mk: Megakaryocytes
Mono: Monocytes
MPP: Multipotent progenitors
mRNA: Messenger RNA
mTOR: Mechanistic targets of rapamycin
My: Myeloid
Neu: Neutrophil
PCA: Principal component analysis
scRRBS: Single-cell reduced representation bisulfite sequencing
SNPs: Single nucleotide polymorphisms
SPADE: Spanning-tree progression analysis of density-normalized events
t-SNE: *t*-distributed stochastic neighbor embedding
UMIs: Unique molecular identifiers
WGA: Whole genome amplification
WGCNA: Weighted gene co-expression network analysis
YS: Yolk sac
